# 

**Published:** 1885-08

**Authors:** 


### CONTENTS.

ORIGINAL ARTICLES: *Pages.*

Electricity in Uterine Diseases. By S. F. Starley, M.D., Ex-Pres. Tex. State Med. Assn., Tyler, 59

Persistent and Alarming Hæmorrhage from the Bladder. By H. C. Ghent, M.D., Ex-Pres. Texas State Med. Assn., Belton, Texas ..... .. 61

Sympathetic Vomiting of Pregnancy. Two cases. One by W. T. Baird, M. D., Albany; one by R. G. Williams, M. D., Whitney ........... 67

How to Cure Some Cases of Acute Eczema. By Q. C. Smith, M. D., Austin .................... 71

An Extraordinary Case in Practice. By R. T. Knox, M. D., Gonzales...................... 72

A Case of Ectrogenesis. By W. J. Burt, M. D., Sec. Texas State Med. Assn., Austin......... 73

CULLINGS FROM CONTEMPORARIES:

Induced Abortion to Relieve Vomiting in Pregnancy 74

Dr. Ferran's Experiments in Cholera Inoculation. 77

Sic-transit,—Another Bubble Burst ........ ..... 79

The Race of Life ............................ 80

Nothing Uncommon........................... 81

A Novel Expedient ........................... 91

CORRESPONDENCE:

Letter from Dr. R. M. Swearingen, Texas State HealthOfficer,on"A Case of Septic-Salpingitis" 83

Letter from Dr. S. A. Greenwell, Cleburne, Texas, Cocaine in Membranous Croup .... ......... 85

Letter of Dr. T. C. Osborn. The Way the Doctors Do in Alabama ............................ 86

SOCIETY NOTES:

Action of Dallas County Medical Society on the International Congress Question............. 89

The Physician's Mutual Benefit Assn. of Texas... 91

EDITORIAL:

Communism in Medicine...................... 92

Et Tu Brute?....... ......................... 96

Mephistophles and the N. Y. Medical Journal.... 99

BIBLIOGRAPHY:

Zeimssen's Handbook of General Therapeutics.... 101

A Text-Book of Hygiene. By Geo. H. Rohe, M.D. 103

MELANGE:

That "Male Uterus" (correction); Give Us the Names; False Hopes; Acknowledgment; Our Next Number ............................ 103

ADVERTISERS' EDITORIAL NOTICES:

The Coming Mecca for Texas Pilgrims; A Valuable Accessory Just Found; Young and Vigorous; Error; The N. Y. Polyclinic; Surgeons Attention; Neurology and Electro-Therapeutics. 105

